# Crystal structure of 2-amino­pyridinium 6-chloro­nicotinate

**DOI:** 10.1107/S2056989015014796

**Published:** 2015-08-12

**Authors:** N. Jeeva Jasmine, A. Rajam, P. Thomas Muthiah, N. Stanley, I. Abdul Razak, M. Mustaqim Rosli

**Affiliations:** a School of Chemistry, Bharathidasan University, Tiruchirappalli 620 024, Tamil Nadu, India; b School of Physics, Universiti Sains Malaysia, 11800 USM, Penang, Malaysia

**Keywords:** crystal structure, 2-amino­pyridinium, 6-chloro­nicotinate, 6-chloro­pyridine-3-carboxyl­ate, noncovalent inter­actions, π–π stacking inter­actions

## Abstract

In the title salt, C_5_H_7_N^+^·C_6_H_3_ClNO^−^, the 2-amino­pyri­din­ium cation inter­acts with the carboxyl­ate group of the 6-chloro­nicotinate anion through a pair of independent N—H⋯O hydrogen bonds, forming an *R*_2_^2^(8) ring motif. In the crystal, these dimeric units are connected further *via* N—H⋯O hydrogen bonds, forming chains along [001]. In addition, weak C—H⋯N and C—H⋯O hydrogen bonds, together with weak π–π inter­actions, with centroid–centroid distances of 3.6560 (5) and 3.6295 (5) Å, connect the chains, forming a two-dimensional network parallel to (100).

## Related literature   

For a background to noncovalent inter­actions, see: García-Raso *et al.* (2009 ▸). For the applications of pyridine compounds, see: Schwid *et al.* (1997 ▸); Rajkumar *et al.* (2015 ▸). For related structures, see: Xie (2007 ▸); Jennifer & Mu­thiah (2014 ▸); Chao *et al.* (1975 ▸); Bis & Zaworotko (2005 ▸); Jebas & Balasubramanian (2006 ▸). For information on π–π stacking inter­actions, see: Hunter (1994 ▸). For hydrogen-bond graph-set motifs, see: Bernstein *et al.* (1995 ▸);
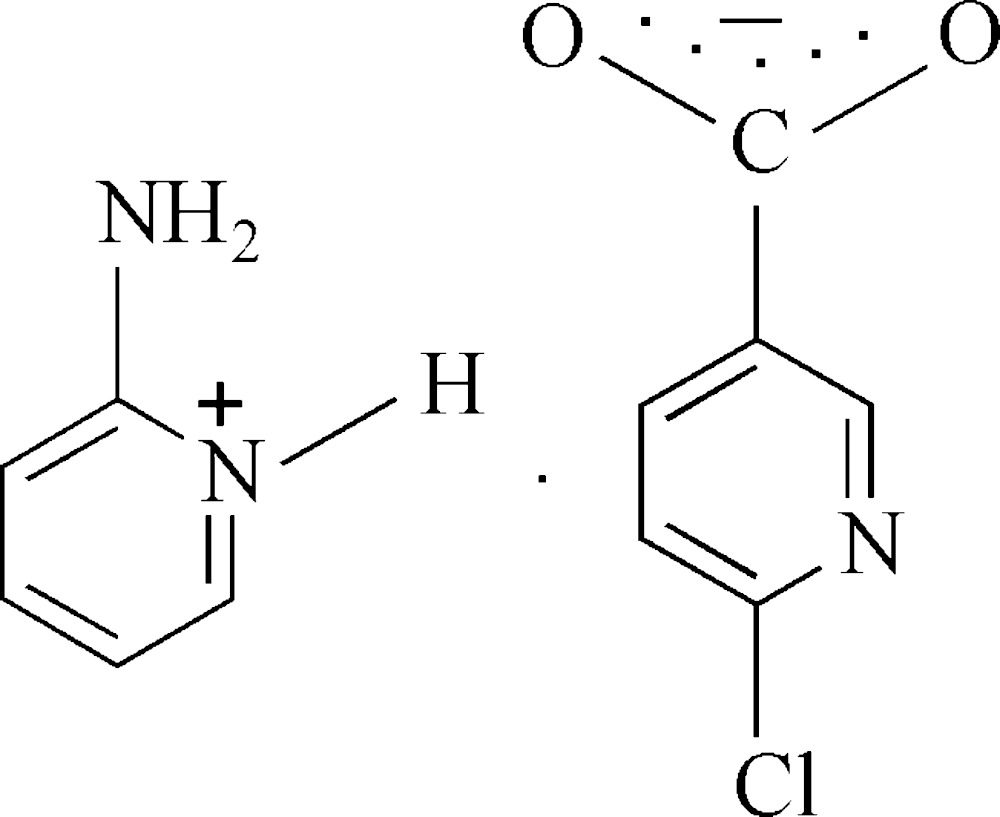


## Experimental   

### Crystal data  

C_5_H_7_N_2_^+^·C_6_H_3_ClNO_2_^−^
*M* *_r_* = 251.67
Monoclinic, 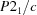

*a* = 8.6844 (4) Å
*b* = 10.8112 (5) Å
*c* = 11.9235 (6) Å
β = 95.2046 (9)°
*V* = 1114.87 (9) Å^3^
*Z* = 4
Mo *K*α radiation
μ = 0.34 mm^−1^
*T* = 100 K
0.51 × 0.40 × 0.17 mm

### Data collection  

Bruker SMART APEXII DUO CCD area-detector diffractometer
Absorption correction: multi-scan (*SADABS*; Bruker, 2009 ▸) *T*_min_ = 0.993, *T*_max_ = 0.994
15546 measured reflections
4073 independent reflections
3771 reflections with *I* > 2σ(*I*)
*R*_int_ = 0.019

### Refinement  

*R*[*F* ^2^ > 2σ(*F* ^2^)] = 0.031
*wR*(*F* ^2^) = 0.092
*S* = 1.07
4073 reflections
166 parameters
H atoms treated by a mixture of independent and constrained refinement
Δρ_max_ = 0.50 e Å^−3^
Δρ_min_ = −0.22 e Å^−3^

### 

Data collection: *APEX2* (Bruker, 2009 ▸); cell refinement: *SAINT* (Bruker, 2009 ▸); data reduction: *SAINT*; program(s) used to solve structure: *SHELXS97* (Sheldrick, 2008 ▸); program(s) used to refine structure: *SHELXL97* (Sheldrick, 2008 ▸); molecular graphics: *PLATON* (Spek, 2009 ▸) and *Mercury* (Macrae *et al.*, 2008 ▸); software used to prepare material for publication: *PLATON*.

## Supplementary Material

Crystal structure: contains datablock(s) global, I. DOI: 10.1107/S2056989015014796/lh5778sup1.cif

Structure factors: contains datablock(s) I. DOI: 10.1107/S2056989015014796/lh5778Isup2.hkl

Supporting information file. DOI: 10.1107/S2056989015014796/lh5778Isup3.cml

**Fig. 1.**
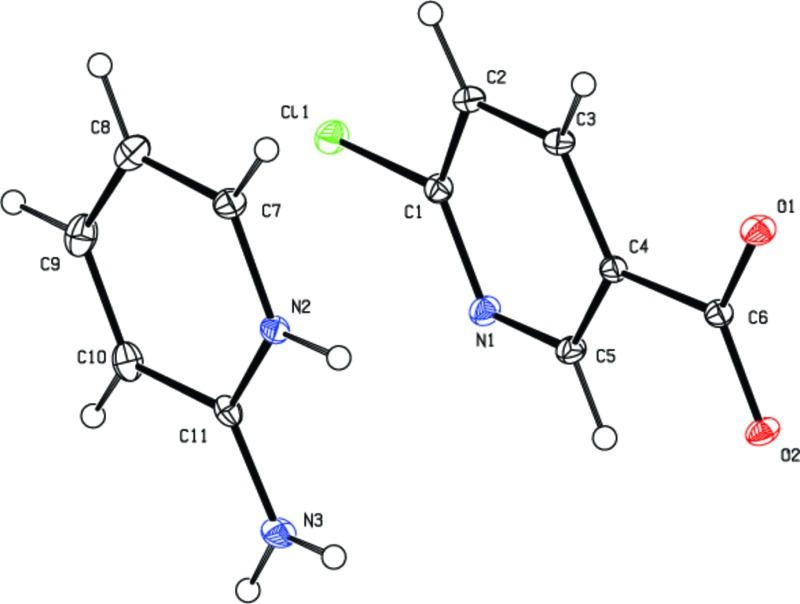
The asymmetric unit of the title compound, showing 30% probability displacement ellipsoids.

. DOI: 10.1107/S2056989015014796/lh5778fig1.tif
The asymmetric unit of the title compound, showing 30% probability displacement ellipsoids.

**Fig. 2.**
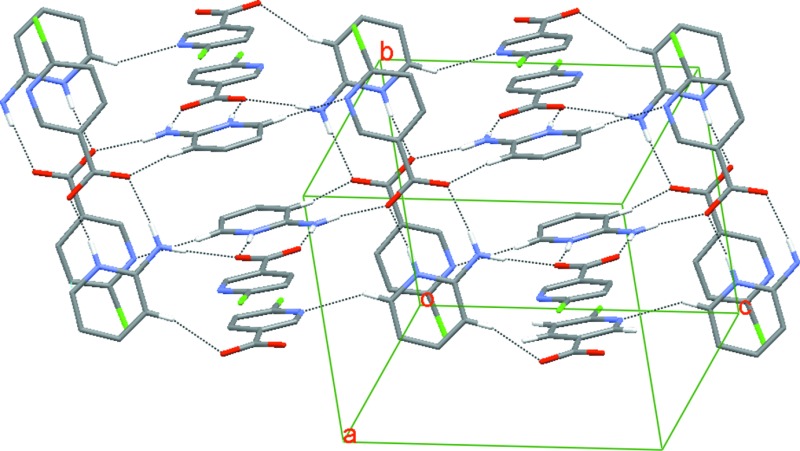
Part of the crystal structure with hydrogen bonds shown as dashed lines. Hydrogen atoms not involved hydrogen bonding have been removed for clarity.

. DOI: 10.1107/S2056989015014796/lh5778fig2.tif
Part of the crystal structure with hydrogen bonds shown as dashed lines. Hydrogen atoms not involved hydrogen bonding have been removed for clarity.

CCDC reference: 1417413

Additional supporting information:  crystallographic information; 3D view; checkCIF report

## Figures and Tables

**Table 1:** Hydrogen-bond geometry (, )

*D*H*A*	*D*H	H*A*	*D* *A*	*D*H*A*
N2H1*N*2O2^i^	0.923(17)	1.781(17)	2.7000(9)	173.5(15)
N3H2*N*3O1^i^	0.844(16)	1.942(17)	2.7830(10)	174.1(15)
N3H1*N*3O2^ii^	0.890(15)	1.962(15)	2.8490(9)	174.0(13)
C7H7*A*N1^iii^	0.95	2.44	3.2808(11)	147
C10H10*A*O1^iv^	0.95	2.25	3.1574(10)	160

Symmetry codes: (i) 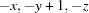; (ii) 
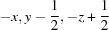
; (iii) 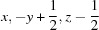; (iv) 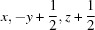.

## supplementary crystallographic information

### S1. Comment

Noncovalent interactions such as hydrogen bonding, anion-π, cation-π, and π-π interactions, and other weak forces play a central role in many areas. They are very important in deciding the conformation of molecules, chemical reactions, molecular recognition, regulating biochemical processes and governing the organization of multicomponent supramolecular assemblies (García-Raso *et al.*, 2009). 2-Aminopyridines are used in the manufacture of pharmaceutical drugs, especially for the treatment of neurological ailments (Schwid *et al.*, 1997). Pyridine heterocycles and their derivatives have large applications in the field of photo-chemical, electrochemical and catalytic process. Some pyridine derivatives possess non-linear optical (NLO) properties (Rajkumar *et al.*, 2015). The crystal structure of 2-aminopyridinium isonicotinate 2-aminopyridine has already been reported (Xie, 2007). The salts of aminopyridine-thiophenecarboxylic acid (Jennifer & Muthiah, 2014) have been recently reported from our laboratory. We report herein the crystal structure of the title molecular salt, obtained by the reaction of 2-aminopyridine with 6-chloronicotinic acid.

The asymmetric unit of the title salt, (I), contains one 2-aminopyridinium cation and a 6-chloronicotinate anion (Fig. 1). Protonation of the cation occurs at N2, providing a C7—N2—C11 angle of 122.45 (7)° compared with 117.7 (1)° in the unprotonated 2-aminopyridine (Chao *et al.*, 1975). A similar type of protonation is observed in various 2-aminopyridine acid complexes (Bis & Zaworotko, 2005). The bond lengths and angles in complex (I) are within normal ranges and comparable to those in other 2-aminopyridinium complexes (Jebas & Balasubramanian, 2006). The carboxylate group of the 6-chloronicotinate anion interacts with the protonated atom N2 and the amino group of the pyridine moiety through a pair of N—H···O hydrogen bonds, forming an eight membered *R*_2_^2^(8) ring motif (Bernstein *et al.*, 1995). Furthermore, these motifs are connected *via* N3—H1···O2^ii^, C7—H7A···N1^iii^ and C10—H10A···O1^iv^ hydrogen bonds (see Table 1 for symmetry codes), forming a two-dimensional network parallel to (100) (Fig 2). The crystal structure is further stabilized by two distinct π–π stacking interactions involving the 6-chloronicotinate and pyridinium ions. A *Cg*1-*Cg*2 distance of 3.6560 (5) Å and *Cg*2—*Cg*2 distance of 3.6295 (5) Å is observed (where *Cg*1 is the centroid of the N1/C1-C5 ring and *Cg*2 is the centroid of the N2/C7-C11 ring). The perpendicular distances of 3.2545 (3) and 3.5411 (3)Å together with the slip angles of 22.3 & 12.7°, respectively are typical for aromatic stacking values (Hunter, 1994).

### S2. Experimental

A hot ethanolic solution of 2-aminopyridine (23 mg, Aldrich) and 6-chloronicotinic acid (39 mg, Alfa Aesar) was warmed for half an hour over a water bath. The mixture was cooled slowly and kept at room temperature. After a few days colourless plate like crystals were obtained.

### S3. Refinement

Hydrogen atoms boned to C atoms were place in calculated postions with with C—H = 0.95Å and included with U_iso_(H) = 1.2U_eq_(C). H atoms boned to N atoms were refined independently with isotropic displacement parameters.

### Crystal data

**Table:**

C_5_H_7_N_2_^+^·C_6_H_3_ClNO_2_^−^	*Z* = 4
*M**_r_* = 251.67	*F*(000) = 520
Monoclinic, *P*2_1_/*c*	*D*_x_ = 1.499 Mg m^−^^3^
Hall symbol: -P 2ybc	Mo *K*α radiation, λ = 0.71073 Å
*a* = 8.6844 (4) Å	θ = 2.4–32.7°
*b* = 10.8112 (5) Å	µ = 0.34 mm^−^^1^
*c* = 11.9235 (6) Å	*T* = 100 K
β = 95.2046 (9)°	Plate, colourless
*V* = 1114.87 (9) Å^3^	0.51 × 0.40 × 0.17 mm

### Data collection

**Table:**

Bruker SMART APEXII DUO CCD area-detector diffractometer	4073 independent reflections
Radiation source: fine-focus sealed tube	3771 reflections with *I* > 2σ(*I*)
Graphite monochromator	*R*_int_ = 0.019
φ and ω scans	θ_max_ = 32.7°, θ_min_ = 2.4°
Absorption correction: multi-scan (*SADABS*; Bruker, 2009)	*h* = −13→13
*T*_min_ = 0.993, *T*_max_ = 0.994	*k* = −16→16
15546 measured reflections	*l* = −18→18

### Refinement

**Table:**

Refinement on *F*^2^	Primary atom site location: structure-invariant direct methods
Least-squares matrix: full	Secondary atom site location: difference Fourier map
*R*[*F*^2^ > 2σ(*F*^2^)] = 0.031	Hydrogen site location: inferred from neighbouring sites
*wR*(*F*^2^) = 0.092	H atoms treated by a mixture of independent and constrained refinement
*S* = 1.07	W = 1/[Σ^2^(*F*O^2^) + (0.0539*P*)^2^ + 0.2679*P*] where *P* = (*F*O^2^ + 2*F*C^2^)/3
4073 reflections	(Δ/σ)_max_ < 0.001
166 parameters	Δρ_max_ = 0.50 e Å^−^^3^
0 restraints	Δρ_min_ = −0.22 e Å^−^^3^

### Special details

**Table:**

Geometry. Bond distances, angles *etc*. have been calculated using the rounded fractional coordinates. All su's are estimated from the variances of the (full) variance-covariance matrix. The cell e.s.d.'s are taken into account in the estimation of distances, angles and torsion angles
Refinement. Refinement on *F*^2^ for ALL reflections except those flagged by the user for potential systematic errors. Weighted *R*-factors *wR* and all goodnesses of fit *S* are based on *F*^2^, conventional *R*-factors *R* are based on *F*, with *F* set to zero for negative *F*^2^. The observed criterion of *F*^2^ > σ(*F*^2^) is used only for calculating -*R*-factor-obs *etc*. and is not relevant to the choice of reflections for refinement. *R*-factors based on *F*^2^ are statistically about twice as large as those based on *F*, and *R*-factors based on ALL data will be even larger.

### Fractional atomic coordinates and isotropic or equivalent isotropic displacement parameters (Å^2^)

**Table:**

	*x*	*y*	*z*	*U*_iso_*/*U*_eq_	
N2	0.08234 (8)	0.17345 (6)	0.03387 (6)	0.0142 (2)	
N3	−0.02920 (9)	0.19691 (7)	0.20096 (6)	0.0184 (2)	
C7	0.17904 (9)	0.11677 (8)	−0.03367 (7)	0.0177 (2)	
C8	0.26497 (11)	0.01655 (9)	0.00290 (8)	0.0228 (2)	
C9	0.25206 (11)	−0.02551 (8)	0.11385 (8)	0.0238 (2)	
C10	0.15609 (10)	0.03247 (8)	0.18212 (7)	0.0197 (2)	
C11	0.06759 (9)	0.13562 (7)	0.14076 (6)	0.0146 (2)	
Cl1	0.54962 (2)	0.18137 (2)	0.23646 (2)	0.0203 (1)	
O1	0.18876 (7)	0.61122 (6)	−0.08641 (5)	0.0192 (2)	
O2	0.07082 (7)	0.63585 (6)	0.07132 (5)	0.0171 (2)	
N1	0.33406 (8)	0.35025 (7)	0.22723 (6)	0.0166 (2)	
C1	0.43180 (9)	0.29272 (7)	0.16629 (7)	0.0150 (2)	
C2	0.44779 (9)	0.31497 (8)	0.05297 (7)	0.0168 (2)	
C3	0.35703 (9)	0.40792 (8)	0.00135 (6)	0.0158 (2)	
C4	0.25507 (8)	0.47360 (7)	0.06340 (6)	0.0129 (2)	
C5	0.24641 (9)	0.43930 (7)	0.17505 (6)	0.0152 (2)	
C6	0.16403 (8)	0.58120 (7)	0.01150 (6)	0.0135 (2)	
H1N2	0.0255 (18)	0.2389 (16)	0.0024 (14)	0.038 (4)*	
H2N3	−0.0831 (18)	0.2538 (16)	0.1688 (13)	0.032 (4)*	
H1N3	−0.0454 (17)	0.1728 (14)	0.2703 (13)	0.031 (4)*	
H7A	0.18690	0.14750	−0.10760	0.0210*	
H8A	0.33130	−0.02370	−0.04470	0.0270*	
H9A	0.31080	−0.09500	0.14140	0.0290*	
H10A	0.14880	0.00380	0.25680	0.0240*	
H2A	0.51770	0.26860	0.01280	0.0200*	
H3A	0.36410	0.42690	−0.07580	0.0190*	
H5A	0.17440	0.48120	0.21690	0.0180*	

### Atomic displacement parameters (Å^2^)

**Table:**

	*U*^11^	*U*^22^	*U*^33^	*U*^12^	*U*^13^	*U*^23^
N2	0.0166 (3)	0.0150 (3)	0.0112 (3)	−0.0003 (2)	0.0027 (2)	0.0014 (2)
N3	0.0217 (3)	0.0218 (3)	0.0125 (3)	0.0003 (3)	0.0053 (2)	0.0033 (2)
C7	0.0201 (3)	0.0187 (3)	0.0148 (3)	−0.0004 (3)	0.0040 (3)	−0.0023 (3)
C8	0.0229 (4)	0.0206 (4)	0.0249 (4)	0.0039 (3)	0.0024 (3)	−0.0044 (3)
C9	0.0256 (4)	0.0174 (4)	0.0274 (4)	0.0034 (3)	−0.0034 (3)	0.0010 (3)
C10	0.0234 (3)	0.0170 (3)	0.0180 (3)	−0.0014 (3)	−0.0024 (3)	0.0050 (3)
C11	0.0166 (3)	0.0150 (3)	0.0121 (3)	−0.0037 (2)	0.0007 (2)	0.0017 (2)
Cl1	0.0195 (1)	0.0189 (1)	0.0224 (1)	0.0050 (1)	0.0020 (1)	0.0033 (1)
O1	0.0249 (3)	0.0212 (3)	0.0123 (2)	0.0042 (2)	0.0063 (2)	0.0025 (2)
O2	0.0212 (3)	0.0187 (3)	0.0119 (2)	0.0056 (2)	0.0047 (2)	0.0000 (2)
N1	0.0196 (3)	0.0161 (3)	0.0145 (3)	0.0028 (2)	0.0036 (2)	0.0008 (2)
C1	0.0144 (3)	0.0138 (3)	0.0167 (3)	0.0005 (2)	0.0016 (2)	0.0004 (2)
C2	0.0164 (3)	0.0175 (3)	0.0173 (3)	0.0017 (2)	0.0060 (3)	−0.0007 (2)
C3	0.0172 (3)	0.0171 (3)	0.0136 (3)	0.0004 (3)	0.0050 (2)	−0.0005 (2)
C4	0.0140 (3)	0.0130 (3)	0.0119 (3)	−0.0008 (2)	0.0025 (2)	−0.0007 (2)
C5	0.0182 (3)	0.0150 (3)	0.0129 (3)	0.0021 (3)	0.0043 (2)	0.0000 (2)
C6	0.0151 (3)	0.0142 (3)	0.0112 (3)	−0.0008 (2)	0.0019 (2)	−0.0008 (2)

### Geometric parameters (Å, º)

**Table:**

Cl1—C1	1.7438 (8)	C10—C11	1.4173 (12)
O1—C6	1.2489 (9)	C7—H7A	0.9500
O2—C6	1.2719 (9)	C8—H8A	0.9500
N2—C11	1.3556 (10)	C9—H9A	0.9500
N2—C7	1.3609 (11)	C10—H10A	0.9500
N3—C11	1.3305 (11)	C1—C2	1.3917 (12)
N2—H1N2	0.923 (17)	C2—C3	1.3863 (12)
N3—H2N3	0.844 (16)	C3—C4	1.3980 (11)
N3—H1N3	0.890 (15)	C4—C5	1.3905 (10)
N1—C5	1.3444 (11)	C4—C6	1.5075 (10)
N1—C1	1.3218 (11)	C2—H2A	0.9500
C7—C8	1.3645 (13)	C3—H3A	0.9500
C8—C9	1.4129 (13)	C5—H5A	0.9500
C9—C10	1.3687 (13)		
			
Cl1···C4^i^	3.5893 (8)	C6···N2^v^	3.4200 (10)
Cl1···C5^i^	3.2804 (8)	C6···O2^v^	3.2056 (10)
Cl1···C9	3.6238 (10)	C7···C3	3.5149 (12)
Cl1···H8A^ii^	3.1000	C7···C2	3.2663 (12)
Cl1···H3A^iii^	3.1000	C7···N1^vii^	3.2808 (11)
Cl1···H9A^iv^	3.0200	C9···Cl1	3.6238 (10)
O1···N3^v^	2.7830 (10)	C10···O1^iii^	3.1574 (10)
O1···C2^vi^	3.2450 (10)	C11···C1	3.5787 (11)
O1···C10^vii^	3.1574 (10)	C11···N1	3.3719 (11)
O2···C6^v^	3.2056 (10)	C3···H3A^vi^	3.0700
O2···C4^v^	3.3415 (10)	C5···H7A^iii^	2.8500
O2···N3^viii^	2.8490 (9)	C6···H1N3^viii^	3.049 (15)
O2···N2^v^	2.7000 (9)	C6···H1N2^v^	2.544 (17)
O1···H3A	2.5000	C6···H2N3^v^	2.833 (16)
O1···H1N2^v^	2.726 (16)	H1N2···H2N3	2.28 (2)
O1···H10A^vii^	2.2500	H1N2···O1^v^	2.726 (16)
O1···H2N3^v^	1.942 (17)	H1N2···O2^v^	1.781 (17)
O2···H5A	2.5200	H1N2···C6^v^	2.544 (17)
O2···H1N2^v^	1.781 (17)	H2N3···O1^v^	1.942 (17)
O2···H1N3^viii^	1.962 (15)	H2N3···C6^v^	2.833 (16)
N1···C11	3.3719 (11)	H2N3···H1N2	2.28 (2)
N1···C7^iii^	3.2808 (11)	H1N3···C6^ix^	3.049 (15)
N2···O2^v^	2.7000 (9)	H1N3···H10A	2.5000
N2···C6^v^	3.4200 (10)	H1N3···O2^ix^	1.962 (15)
N3···O1^v^	2.7830 (10)	H1N3···H5A^ix^	2.3700
N3···O2^ix^	2.8490 (9)	H3A···O1	2.5000
N1···H7A^iii^	2.4400	H3A···C3^vi^	3.0700
N3···H5A^ix^	2.8700	H3A···Cl1^vii^	3.1000
C1···C11	3.5787 (11)	H5A···O2	2.5200
C2···C3^vi^	3.5309 (12)	H5A···N3^viii^	2.8700
C2···C7	3.2663 (12)	H5A···H1N3^viii^	2.3700
C2···O1^vi^	3.2450 (10)	H5A···H7A^iii^	2.5100
C3···C3^vi^	3.1853 (12)	H7A···H5A^vii^	2.5100
C3···C7	3.5149 (12)	H7A···N1^vii^	2.4400
C3···C2^vi^	3.5309 (12)	H7A···C5^vii^	2.8500
C4···Cl1^iv^	3.5893 (8)	H8A···Cl1^ii^	3.1000
C4···O2^v^	3.3415 (10)	H9A···Cl1^i^	3.0200
C5···Cl1^iv^	3.2804 (8)	H10A···O1^iii^	2.2500
C6···C6^v^	3.3366 (10)	H10A···H1N3	2.5000
			
C7—N2—C11	122.45 (7)	C9—C10—H10A	120.00
C7—N2—H1N2	116.2 (10)	C11—C10—H10A	120.00
C11—N2—H1N2	121.4 (10)	Cl1—C1—N1	116.05 (6)
C11—N3—H2N3	118.0 (11)	Cl1—C1—C2	118.64 (6)
C11—N3—H1N3	121.1 (10)	N1—C1—C2	125.31 (7)
H2N3—N3—H1N3	120.5 (14)	C1—C2—C3	116.97 (7)
C1—N1—C5	116.58 (7)	C2—C3—C4	119.68 (7)
N2—C7—C8	121.16 (8)	C3—C4—C5	117.59 (7)
C7—C8—C9	117.85 (8)	C3—C4—C6	120.56 (6)
C8—C9—C10	120.92 (8)	C5—C4—C6	121.80 (6)
C9—C10—C11	119.58 (8)	N1—C5—C4	123.81 (7)
N3—C11—C10	123.60 (7)	O1—C6—O2	125.14 (7)
N2—C11—N3	118.37 (7)	O1—C6—C4	117.13 (6)
N2—C11—C10	118.04 (7)	O2—C6—C4	117.71 (6)
N2—C7—H7A	119.00	C1—C2—H2A	122.00
C8—C7—H7A	119.00	C3—C2—H2A	121.00
C9—C8—H8A	121.00	C2—C3—H3A	120.00
C7—C8—H8A	121.00	C4—C3—H3A	120.00
C8—C9—H9A	120.00	N1—C5—H5A	118.00
C10—C9—H9A	120.00	C4—C5—H5A	118.00
			
C11—N2—C7—C8	1.11 (12)	Cl1—C1—C2—C3	−177.22 (6)
C7—N2—C11—N3	179.67 (8)	N1—C1—C2—C3	2.22 (13)
C7—N2—C11—C10	−0.50 (11)	C1—C2—C3—C4	−0.29 (12)
C1—N1—C5—C4	−0.78 (12)	C2—C3—C4—C5	−1.86 (11)
C5—N1—C1—Cl1	177.76 (6)	C2—C3—C4—C6	175.44 (7)
C5—N1—C1—C2	−1.69 (12)	C3—C4—C5—N1	2.51 (12)
N2—C7—C8—C9	−0.90 (13)	C6—C4—C5—N1	−174.75 (7)
C7—C8—C9—C10	0.14 (14)	C3—C4—C6—O1	−2.82 (11)
C8—C9—C10—C11	0.44 (13)	C3—C4—C6—O2	178.64 (7)
C9—C10—C11—N2	−0.26 (12)	C5—C4—C6—O1	174.36 (7)
C9—C10—C11—N3	179.55 (8)	C5—C4—C6—O2	−4.18 (11)

Symmetry codes: (i) −*x*+1, *y*−1/2, −*z*+1/2; (ii) −*x*+1, −*y*, −*z*; (iii) *x*, −*y*+1/2, *z*+1/2; (iv) −*x*+1, *y*+1/2, −*z*+1/2; (v) −*x*, −*y*+1, −*z*; (vi) −*x*+1, −*y*+1, −*z*; (vii) *x*, −*y*+1/2, *z*−1/2; (viii) −*x*, *y*+1/2, −*z*+1/2; (ix) −*x*, *y*−1/2, −*z*+1/2.

### Hydrogen-bond geometry (Å, º)

**Table:**

*D*—H···*A*	*D*—H	H···*A*	*D*···*A*	*D*—H···*A*
N2—H1*N*2···O2^v^	0.923 (17)	1.781 (17)	2.7000 (9)	173.5 (15)
N3—H2*N*3···O1^v^	0.844 (16)	1.942 (17)	2.7830 (10)	174.1 (15)
N3—H1*N*3···O2^ix^	0.890 (15)	1.962 (15)	2.8490 (9)	174.0 (13)
C7—H7*A*···N1^vii^	0.95	2.44	3.2808 (11)	147
C10—H10*A*···O1^iii^	0.95	2.25	3.1574 (10)	160

Symmetry codes: (iii) *x*, −*y*+1/2, *z*+1/2; (v) −*x*, −*y*+1, −*z*; (vii) *x*, −*y*+1/2, *z*−1/2; (ix) −*x*, *y*−1/2, −*z*+1/2.
