# Supplementary figures and images for: Crystal structure of 2-amino­pyridinium 6-chloro­nicotinate
Source: Acta Crystallogr E Crystallogr Commun. 2015 Aug 12;71(Pt 9):o655–6. doi: 10.1107/S2056989015014796 (PMC4555436; doi:10.1107/S2056989015014796)

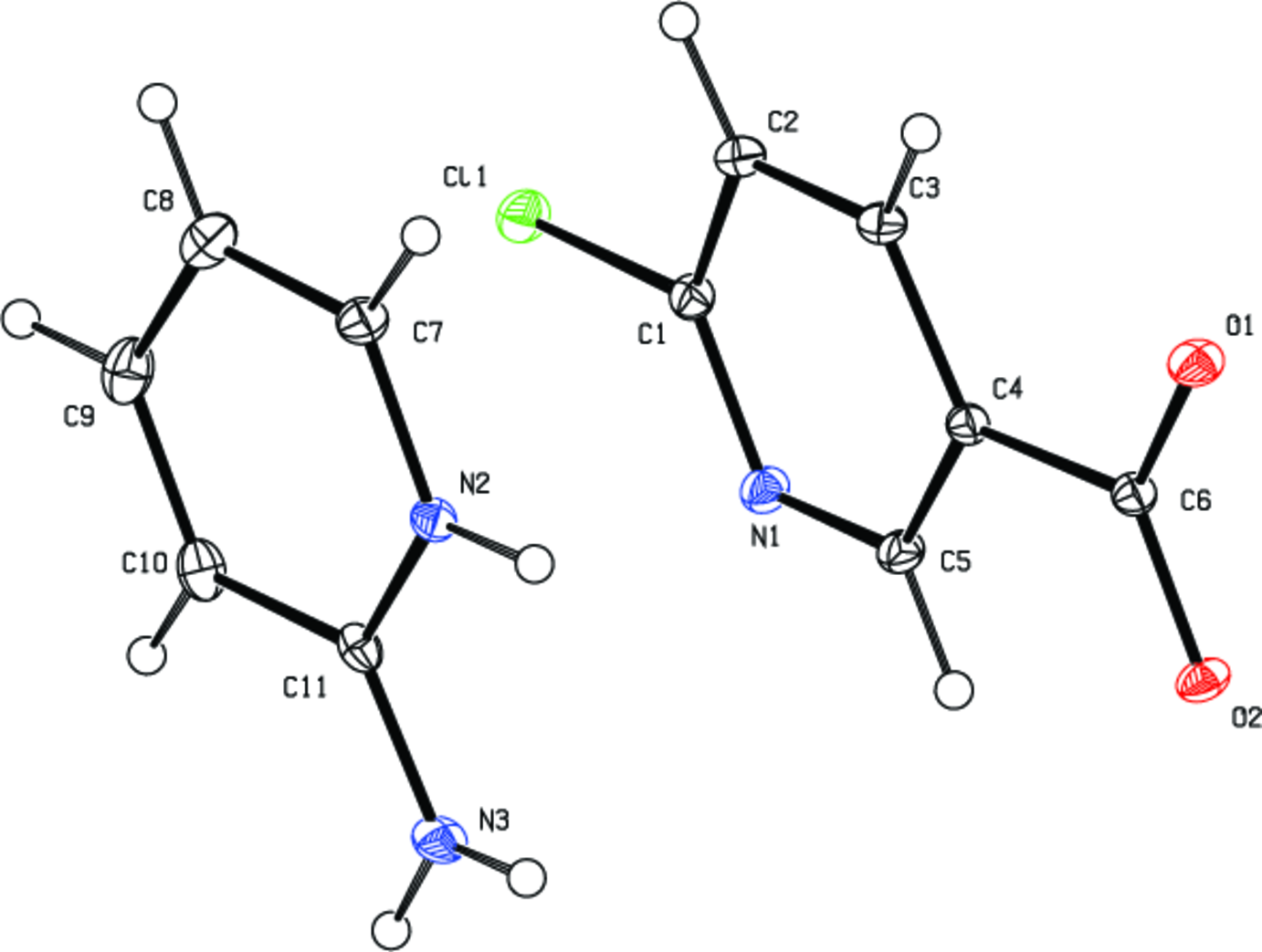

Supplement: Supplementary file 4 [file e-71-0o655-fig1.tif]

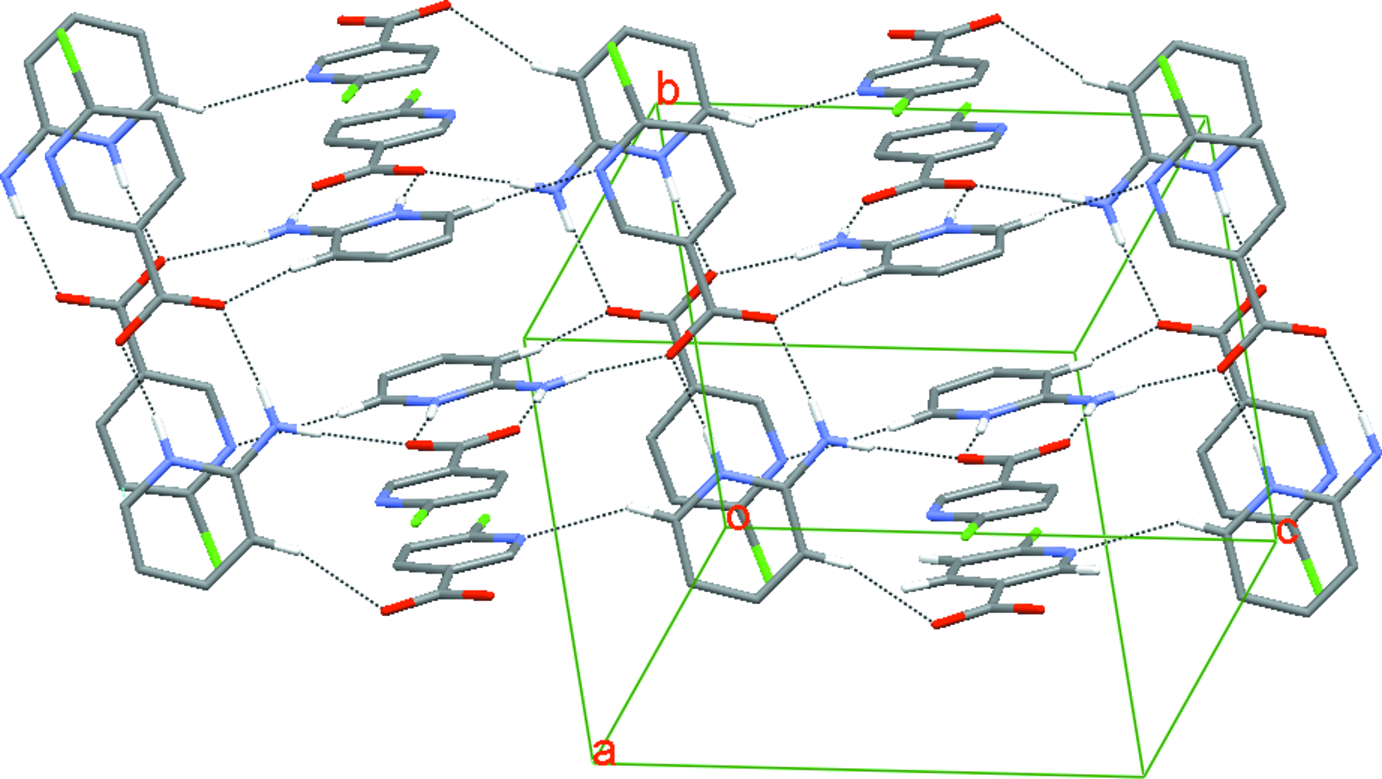

Supplement: Supplementary file 5 [file e-71-0o655-fig2.tif]
